# Hospital Reports

**Published:** 1837

**Authors:** 


### WESTERN JOURNAL

OF THE

### MEDICAL AND PHYSICAL SCIENCES:

**For July, August, and September, 1837.**

### ESSAYS AND CASES.

### Art I.—Hospital Reports.

*Cases treated in the* Cincinnati Hospital; *with Clinical observations.* Recorded by Edward Kimball, House Surgeon. Arranged by the Editorial Committee.

### CLINICAL DEPARTMENT.

#### Apparent Delirium Tremens.—Death.—Dissection.

Case.—J. K., a marine, aged 21, was admitted into the Hospital, on the 30th of January. His habits were intemperate, and for several days before he was taken ill, had frequently wet his feet. His attack came on with rigors, headache, vomiting, diarrhœa, epigastric tenderness, a slight cough, and some fever. When brought to the Hospital, his respiration was hurried, and his expression wild and anxious, with considerable engorgement of the eyes. When received into the institution, it was evening. He was directed to lose eight ounces of blood, and take ten grains of calomel, with five of Dover's powder. At eleven o'clock, being restless, two grains of solid opium were administered.

31st—Morning.—Diarrhœa suspended; nausea and retching continuing; tongue dry and red; pulse above 100, feeble; restlessness considerable; delirium, with hurried respiration.

Directed to take opium and camphor. Afternoon—Pulse increased in frequency ; delirium greater and more characteristic. Took an emetico-cathartic, which operated well, but afforded no relief. At night quite wild, with a strong inclination to escape ; pulse very feeble ; breathing quick and laborious ; skin shrunken ; occasionally comatose. Ordered wine, with ammoniated alcohol and laudanum, each thirty drops every hour, with a blister to the nucba. Pulse rose a little, but the delirium increased, and he made repeated attempts to escape. Towards morning, became more composed, and desired the nurse to leave the room on an errand, when he arose from his bed, and sprang through the sash of one of the windows of his chamber, before the inmates of the ward could arrest him. Falling about fourteen feet on the wooden steps of a door, he was but slightly stunned, and suffered no local injury, but the dislocation of the first bone of one of his toes. Being restored to his bed, he was directed to take a hundred drops of laudanum, every two hours, till he became composed. At 2 o'clock, P. M., he was partially narcotissed, and the medicine was discontinued. At 9, P. M., having become more watchful, opium in two grain doses, with calomel, was ordered, of which he took several doses, with wine. Symptoms increasing, he expired the next evening.

### Examination of the Body—By Professor Gross.

Brain.—The vessels of the different membranes very much injected with blood, with some sero-fibrous effusion upon them. Medullary matter firm, and so much congested, as to bleed when cut into—cineritous substance very dark—cerebellum considerably injected.

Thorax.—Effusions of coagulating lymph upon the pleura. Heart preter-naturally large, with its substance uncommonly vascular. Pericardium deeply injected, with a yellowish synovial fluid in its cavity. Lungs, with their mucous membrane injected ; false membrane on the posterior and upper part of the right lung, its parenchyma engorged with blood ; partial hepatization ; small quantities of purulent secretion.

Abdomen.—Stomach with red streaks along its rugæ ; bowels contracted ; duodenum and illium slightly reddened ; spleen rather large, otherwise natural ; liver and gall bladder healthy ; pancreas sound.

### Delirium Tremens, again.

Case.—This was a well-educated, and once respectable man, of about 35 years of age, who after a somewhat protracted frolic, was brought by his friends to the Cincinnati Hospital. He had decided manifestations of mania a potu, which grew worse, till he became so wild and uncontrollable, as to require the straight jacket. There was no appearance of any inflammatory irritation in the nervous centres, or in the mucous passages, but an erethismus of a peculiar nature, occupying the powers of innervation. He slept not for several nights, and made frequent attempts to escape by the window and door of his room. He recovered. An account of the treatment will appear at the close of the following:

### Clinical Remarks.

Professor Harrison, in a brief commentary on his case, took occasion to advert to the pathology and treatment of this singular affection. He successively reviewed the theories of Broussais and others, who respectively localize the disease in the mucous coat of the alimentary canal, in the encephalon, or in blood. He pointed out three stages, as evinced by the disease; first, a stage of incubation, marked by diminished nervous, muscular and vascular power, the stage of depression; second, the stage of increased excitement, characterized by a high display of sensorial disturbance, muscular energy, and vascular commotion; and third, that of collapse.

The Professor contended that there existed no satisfactory proof of the essential inflammatory nature of the affection; but admitted that such a complication as that of inflammation of the brain, or stomach, might be present in some instances, though such cases were unfrequent.

Delirium tremens is not a simple state of depressed nervous energy, nor does it consist in a mere augmentation of the powers of innervation. It is not an *exaltation* of a physiological state, but a *perversion* of it; and this is evinced by the wild and disordered train of thought and feeling, connected with the incessant muscular activity which invariably attend his complaint. Prof. H. designated this peculiar state of the nervous energy, an erethism of the organic, or ganglionic system of nerves, which involved the sensorial functions in its sympathetic irradiations. The indication of cure, was to calm this erethism of the ganglionic nervous apparatus. Venesection might be proper in a few instances to subdue vascular action, where inflammation of the brain, or abdominal organs impended; but as a general rule of practice, he urged that bleeding was not required. The plan of treatment of Dr. Klapp, of Philadelphia, that of emetics, is valuable—for independently of the evacuant effects achieved by the vomit employed, it is certain that a soothing and tranquilizing influence is exerted on the nervous energies by the remedy.

The cold dash on the head, or over the entire body, may be useful in subduing the enormous wildness of the patient, and in reducing the agitated movements of the innervation. But, says Prof. H., I wish to put you on your guard in reference to the cold bath, for I once lost a patient under its operation in this disease.

Calomel you will find it proper to administer in conjunction with opium, or some other narcotic, to fulfil the subordinate indication prescribed in most of such attacks, that is, the restoration of the secretions. But independent of the important end thus subserved, by our mercurial medicines, the free and increased secretions induced by the calomel, materially aid in conducting off the irritation of the innervation, so conspicuously present in a seizure of temulent mania.

The indication then is to bring about a quietude of the erethism of the ganglionic apparatus of nerves. To attain this, bleeding *may be* demanded, emetics *are often called for*, calomel is *frequently* indicated, *but our main trust must be reposed in opium.* You, gentlemen, says Prof. H. see to what extent the use of this great anodyne may be carried. This man has taken subsequently to the operation of the emetic which we gave him, and which procured a pretty full result in the evacuation of the stomach, and the secretion and ejection of bile, two grains of opium, two of camphor, and two and a half of calomel every two hours, till twenty four grains of opium, the same quantity of camphor, and thirty grains of calomel, were given. He has taken upwards of seven hundred drops of laudanum, with twelve drachms of Hoffman's anodyne—which remedies have procured sleep, after persevering in their use for the last forty eight hours.

If you wish to open the bowels, or freely purge your patient, which is sometimes a good practice, then, instead of opium, give the following:

℞ Camphor ℈ij.
Extr. Hyoxyamus ℈j.
Gum Arabic ʒij.
Water ℥viij.—*Mix.*

A table spoonful to be given every two hours, whilst you are administering calomel and jalap, or other purgative medicines; but your main reliance is to be placed on opium. I prefer, continued the Professor, opium to morphine; it is more stimulating, and acts upon the cuticular circulation more energetically—it is therefore preferable to its preparation in this affection.

### Chronic Diarrhœa.—Softening and Ulceration of the Mucous Membrane of the Intestines.

Case.—H. P. E., a gentleman from Alabama, was brought to the Hospital, May 31, 1837, in a very advanced state of disease. He was 22 years old, and had resided for the last 3 years in the South. He was so much reduced, that no very intelligible history of the past could be collected from him, but it appeared that his habits were temperate ; that early last winter he had a severe and tedious catarrh ; that in February he was seized with dysentery terminating in diarrhœa ; that the intestinal symptoms had remained upon him to the present time, but the pectoral, with the exception of the dyspnœa, had ceased.

His present symptoms are great emaciation, debility, partial loss of power in the lower extremities—says they are sore and hot, though they actually feel cold to the hand—nausea; abdominal pain, especially in the umbilical region; alvine evacuations frequent, light colored and watery; urine free and transparent; voice reduced to a husky whisper; tongue pale, red and glossy; great thirst; pulse 105 in a minute, weak and quick; skin cool and dry. Professor Rives prescribed for him different remedies, internally and externally, which need not be enumerated, as nothing could of course be expected from them, and he died on the sixth day after his admission.

Professor Gross examined the body, in presence of his colleagues and the pupils, and reported the following morbid appearances:

Body, in a state of atrophy.

Lungs.—Interlobular emphysema, with adhesion to the pleura of the superior and posterior part of the left lung, in which was found one large tubercle, the other parts sound.

Heart and pericardium healthy.

Omentum and peritoneum, natural.

Liver, healthy, but the gall bladder much distended, projecting an inch and a half below the edge of the liver, and containing six ounces of dark inspissated bile; ducts unobstructed.

Spleen, of a natural size and healthy color, but containing many osseous concretions; pancreas healthy.

Mesenteric glands much enlarged, and the veins of the mesentery greatly increased in size.

Œsophagus, not inflamed, but its mucous glands enlarged.

Mucous coat of the stomach softened. Mucous coat of the duodenum in a state of inflammatory hyperæmia, and softened, its glands enlarged and hardened. The mucous membrane of the ilium, in a state of inflammation, and softened, with many dark purple spots, one of which verging to gangrene. The glands of Peyer and Brunner slightly enlarged. Colon, signs of inflammation, with softening throughout its whole extent, but greatest in its sigmoid flexure, where there was a large ulcer which had penetrated to the peritoneal coat.

Kidneys and bladder, natural.

Brain, not examined, as the patient displayed no signs of cerebral disease.

### Typhoid Stage of Bilious Fever.

Case.—Lewis Bowman, a young German, 18 years of age, was admitted into the Hospital, June 30th, 1837. He is of delicate structure, and rather small stature, but has enjoyed good health previous to this febrile attack. Has been occupied in one of the many steam boats which navigate the Ohio and Mississippi, and necessarily much exposed as a common work hand.

Soon after leaving New Orleans, from which city the boat to which he is attached, has very recently come, Lewis was seized with slight premonitory symptoms of fever—such as erratic pains, loss of appetite, disturbed sleep, &c. On the boat, 22d June, he took a tea'spoonful of calomel ; this was administered by one of the officers of the vessel, there being no physician aboard. This dose brought on, or was succeeded by, frequent discharges of a dysenteric character, accompanied with tormina and tenesmus. On the 25th he arrived at this port, and sent for a physician, who bled him, applied a blister over his stomach, and gave him more calomel, determining it to prompt cathartic results, by castor oil. He now had high fever, with a persistence of the dysenteric symptoms. Having been treated with repeated doses of calomel, and auxiliary medicines of a purgative character, and his skin having been spunged with iced water, during the height of the fever, there followed some mitigation of the febrile orgasm. On his reception into the hospital, the following phenomena were present in his case. Hot skin, pain and great tenderness about the scrobiculus cordis, flushed cheeks, anxious countenance, tongue coated, and red along its edges, deficient moisture of the mouth, sordes about the teeth, eyes injected, pulse 120, and without hardness, bowels affected with frequent watery, inodorous discharges, and slightly tympanitic, and there is perceptible tendency to coma.

He was ordered by the attending physician, Professor Harrison, half an ounce of Spiritus Mindereri, with thirty drops of Sp: Vit. Dulc. every two hours.

1st. July. Very little deviation in the symptoms—more manifestation of sensorial disturbance. Ipicac, to the amount of one grain, was added to yesterday's prescription.

2d. Medicine has produced frequent thin alvine evacuations, there is more coma, pulse is more rapid. Some subsultus tendinum is present. The mixture was discontinued, and three grs. Dover's powder, and half a grain of camphor were given every three hours.

3d. The frequency of the alvine discharges was arrested. The mixture was resumed.

4th. The symptoms worse, bowels very much disturbed, pulse more frequent, skin still hot, tongue much coated and dry. Discontinue mixture, resume the powders, sponge the surface with vinegar and water. The surface of the abdomen was cupped on the 5th, and antimonial powder given—aided in its action on the skin by a weak decoction of the eupatorium perfoliatum. But these medicines were omitted after he had taken a few doses, as they induced frequent watery evacuations from the bowels. But a few ounces of blood were obtained by the cupping. Calomel, Dover's powder, and camphor were given, one fourth of a grain of the first, and the relative quantities of the other two as stated above.

6th. He is sinking—subsultus, lies on his back, pulse small and frequent, skin becoming cold, bedewed with a clammy perspiration, bowels affected with meteorism. The volatile julep was given with laudanum, and blisters were applied to the ankles.

7th. Omit the vol. julep, and give the hydrar: cum creta, two and a half grains, with one sixth of morphia every four hours, wine and water, chicken water, to be frequently given in small quantities.

8th. Worse. Apply large blister over abdomen, and dress it with strong mercurial ointment. The patient seems to be moribund, pulse scarcely perceptible, skin cold, with clammy sweat, eyes half open, and shewing their white portions only. Two grains of opium were given at bed time—this seemed to have a very good effect.

9th. Quinine, two grs. and opium half a grain, with three fourths of a grain of camphor, were given every two hours.—Blisters were dressed with mercurial ointment.

10th. Better. Give weak chicken soup, wine and water, and one and a half grains of opium at bed time, the powders to be continued.

Lewis went on improving for several days, then became worse—the pulse was exceedingly feeble on the 13th, bowels much disturbed, skin cold. On this day, one grain of opium, and two of camphor, were given every two hours with snake-root tea, which brought up the system, and arrested the discharges from the bowels. Brandy was given in sago on the 14th, and the two grains of opium given at bed time.

On the 16th, salivation commenced, and as he increased in strength, the mercurial action became fully developed.

On the 20th, his appetite was pretty good; the stimulants were gradually withdrawn, and a light nourishing diet allowed.

On the 21st, he was seized with delirium, and could scarcely be kept in bed. This was checked by a resumption of the stimulants—porter was ordered, a wine glassfull every three hours, and opium all night, and no solid aliment was permitted him. In a few days the delirium left him, but his mind is yet weak and childish.

August 2d, is out of bed, has a good appetite, and takes no medicines.

Professor Harrison, in commenting on this case, told the clinical class, that it was valuable for the light it shed on two very interesting points of therapeutics. The first point illustrated by the case, was that which the sagacious mind of Sydenham had discerned, which is, that delirium is caused by opposite states of the encephalon as regards its vascular action. The delirium of Lewis, which urged him to a determined effort to leave his bed and the house, after the febrile symptoms had disappeared, was of an atonic kind, and remediable by mild nutritive incitants. Porter, or good bottled ale, the Professor prefers, in such cases, to wine.

The second point of great practical import, illustrated by this case, was the very much debated question, of the true indication of treatment in the last stage of fever. Broussais, says Prof. H. tells us, that inflammatory irritation of the mucous surface of the alimentary tube, exists in all the varieties and stages of fever. Louis contends that follicular ulceration exists in fever. The plan of treatment in fever, as grounded on either of these theories—even if the theories were true, which we will not now pause to examine—is not necessarily that pursued by Broussais, namely, gum water and leeches.—For we are well assured by a just experience, that inflammation is not to be treated in all its varieties or stages, in a similar manner.

Pathological anatomy, says Prof. H. is exceedingly valuable in conducting the mind to correct conclusions in reference to the prognosis and diagnosis of fever, but is not always successful in suggesting a sound febrile therapeutics.

M. Chomel, a distinguished clinical professor of the School of Medicine of Paris, in a late work entitled "Lecons de Clinique Medicale," has most clearly shown, that under a tonic and stimulant plan in the latter stages of fever, follicular ulceration is more tractable, and tends to a speedier restorative action, than under the opposite mode of treatment. I am aware, says the Professor, that a very nice distinction has been attempted between the morbid anatomy of typhus and typhoid fever. This distinction is not to be relied upon. I cannot rely on it, and I advise you, gentlemen, not to permit your minds to be misled by such refined, or transcendental pathology. Fever is an idiopathic affection, and is variously modified by many collateral circumstances. The first stage of fever is that of lesion of innervation; the second that of lesion of secretion; the third stadium is characterized by a superinduction of vascular irregularities on the lesions of nervous function, and of secretory action; and the fourth stage is that of local irritations, and structural lesions.

Our indications in fever are, first, to abate vascular activity; second, restore the secretions, and by revulsive medication, and the alterative agency of appropriate remedies, to relieve the affected organs, and break up the catenated train of morbid processes, going in the organism.

In the typhoid stage of bilious fever, our indications of treatment, as legitimately established, on the mixed state of nervous, vascular, and secretory irregularities, are to quiet the irritability of the system; restore in a very gentle manner, the secretions; and arrest the lesions which may be set up, and destructively advancing, in the mucous surface of the intestinal tube, or of the bronchial apparatus, or in the great nervous centres. This we accomplish by tonic, incitant, anodyne remedies, by counter irritation to the surface, and by the alterant agency of mercury. This last is much abused in the latter stages of fever. Prof. H. entered his solemn protest against that blind and bold practice, which urges to the administration of scruple after scruple, and drachm after drachm, of calomel, in fever, especially in the typhoid stage.

There is nothing novel, continued the Professor, in the practice pursued in the above case; it is a practice long known and acted upon by American physicians—and now there comes a voice of corroboration from the very fountain head of pathological science in Europe, which testifies to the truth of that sound discriminating clinical judgment, for which our countrymen are so distinguished, in their encounters with disease.

PRESUMED TYPHOID FEVER.—FATAL TERMINATION.—DISSECTION.

CASE.—On the 21st of August, in the afternoon, J. Williams, a marine, was brought to the Hospital. Very little could be learned of his illness, but it appeared that he had been taken down with a fever, ten days before, as the boat to which he was attached came up the Mississippi. He had not been bled, and had taken no medicine, except two doses of cathartic pills.

When brought to the Hospital, he was exceedingly prostrate, with confusion of mind, and had a slow, feeble pulse, cool skin, and dry, brown, cracked tongue. Ordered Dover's powder and calomel in five grain doses, mixed. The next day his skin was hot, and his pulse frequent. A large blister applied to the abdomen, drew well, but he declined rapidly, becoming blind and apparently deaf. In 36 hours after entering the Hospital, he expired.

*Post Mortem* examination on the morning of the 28th, by Professor Harrison.

BODY muscular, not emaciated, tinged with yellow. On opening the cavities, the external appearance of the VISCERA, thoracic and abdominal, was natural.

THORAX.—Lungs natural, except some bony or calcareous deposits in the right lobe. Heart natural—liquor pericardii about one ounce.

ABDOMEN.—Stomach large and distended with gases, and a dark, dirty, green fluid. Mucous membrane coated with a copious secretion of mucus—not softened; several large spots of bright red hyperæmia; blood not removable from them by pressure; sub-mucous capillaries injected under those spots; no ulceration.

Bowels.—Duodenum, containing yellow bile; valvulæ conniventes considerably congested; also sub mucous vessels; no softening of the mucous coat. Jejunum, ilium, cœcum, and colon, with general capillary fulness, diffused and equally inclining to purple, apparently from previous plethora; glands of the ilium, both solitary and accumulated, sound; no follicular ulceration; no softening of the mucous membrane.

Pancreas sound; also the spleen, though rather large. Liver sound, large, not much blood in it; rather dark; gall bladder two-thirds full of dark greenish, grumous bile; mesenteric glands sound; omentum emaciated.

Urinary bladder empty and firmly contracted; kidneys sound. Semi lunar ganglion and great sympathetic sound.

Brain.—Substance and membranes sound; no effusion; not engorged.

Professor Harrison, directed the minds of the clinical students to this dissection, as illustrative of his previous remarks on fever. Here we find no adequate organic lesion to account for the patient's death. The febrile orgasm seems to have imparted a shock to the nervous system which induced the train, or catenation, of phenomena present in the case. The patient died delirous—lost his sight before death, and to a partial extent, his hearing—was nearly spechless for several days, before his admission into the Hospital. There was no no difficulty in procuring alvine discharges—the liver secreted bile in abundance, and of a healthy aspect—the blister applied on the day he died, to the abdomen, drew pretty well.—There was no dothinenteritis, or follicular ulceration—no inflammation of the brain—none of the arachnoid membrane—none of the semilunar ganglion, or of the great sympathetic. Why then should he have died?—The limited extent of vascular fulness, arising from inflammatory irritation, found in the stomach and duodenum, would not constitute a just ground of the true pathological character of the case, unless another, and higher element of morbid action was involved. That element was the great constitutional disturbance—the lesion of innervation—always in effective play in idiopathic fever—and constituting its primary link..

